# A global dataset of crowdsourced land cover and land use reference data

**DOI:** 10.1038/sdata.2017.75

**Published:** 2017-06-13

**Authors:** Steffen Fritz, Linda See, Christoph Perger, Ian McCallum, Christian Schill, Dmitry Schepaschenko, Martina Duerauer, Mathias Karner, Christopher Dresel, Juan-Carlos Laso-Bayas, Myroslava Lesiv, Inian Moorthy, Carl F. Salk, Olha Danylo, Tobias Sturn, Franziska Albrecht, Liangzhi You, Florian Kraxner, Michael Obersteiner

**Affiliations:** 1 Ecosystems Services and Management Program, International Institute for Applied Systems Analysis (IIASA), Laxenburg A-2361, Austria; 2 FeLis, Albert Ludwigs University of Freiburg, Freiburg D-79106, Germany; 3 Southern Swedish Forest Research Center, Swedish University of Agricultural Sciences, Alnarp SE-230 53, Sweden; 4 GeoVille Information Systems GmbH, Innsbruck A-6020, Austria; 5 Environment and Production Technology, International Food Policy Research Institute (IFPRI), Washington, District Of Columbia 20005, USA; 6 Key Laboratory of Agri-informatics, Ministry of Agriculture/Institute of Agricultural Resources and Regional Planning, Chinese Academy of Agricultural Sciences, Beijing 100081, China

**Keywords:** Environmental chemistry, Geography

## Abstract

Global land cover is an essential climate variable and a key biophysical driver for earth system models. While remote sensing technology, particularly satellites, have played a key role in providing land cover datasets, large discrepancies have been noted among the available products. Global land use is typically more difficult to map and in many cases cannot be remotely sensed. *In-situ* or ground-based data and high resolution imagery are thus an important requirement for producing accurate land cover and land use datasets and this is precisely what is lacking. Here we describe the global land cover and land use reference data derived from the Geo-Wiki crowdsourcing platform via four campaigns. These global datasets provide information on human impact, land cover disagreement, wilderness and land cover and land use. Hence, they are relevant for the scientific community that requires reference data for global satellite-derived products, as well as those interested in monitoring global terrestrial ecosystems in general.

## Background & Summary

As an essential climate variable and a key biophysical driver, global land cover is an important baseline dataset^1^. Land cover is defined as the observed biophysical cover that can be found on the surface of the Earth^2^. Earth observation satellites in particular have played a key role in providing land cover information, yet large spatial discrepancies have been noted among the available maps and products^3,4^. These discrepancies mean that the use of one dataset versus another will have considerable implications for results derived from models that depend upon these data^5^. One reason for the growing disparity among new datasets is the lack of suitable reference data across much of the Earth’s surface, a key requirement for producing accurate land cover datasets.

Global land use, which is a fundamental driver of environmental change, is much more difficult to map than land cover and in many cases cannot be directly sensed from satellite imagery. Land use can be broadly defined as the manner in which the observed biophysical cover is actually used by humans^6^. Thus, timely reference data are a crucial input for producing reliable maps of land use.

Proposed international land cover reference data standards^7,8^ have resulted in new datasets such as the global land cover validation reference dataset^8,9^. However, sample sizes for global land cover and land use reference datasets are relatively small—on the order of several hundred to several thousand samples^10^. Cost and practical problems posed by the assessment of global maps have limited the efforts to assess products at global scales^8^. To address the lack of reference data, crowdsourcing has been shown to provide accurate, timely and cost-effective data complementing traditional methods of data acquisition^11,12,13^. Here we describe the global land cover and land use data collected using the Geo-Wiki (http://geo-wiki.org/) crowdsourcing platform from four separate campaigns. The Geo-Wiki platform helps registered users to visualize existing spatial information such as land cover maps, overlaid upon high to medium resolution satellite imagery. Furthermore, it can be used to train people in visual classification, who are then assigned various image interpretation tasks at specific locations around the world for the collection of land cover and land use information.

**Figure 1:**
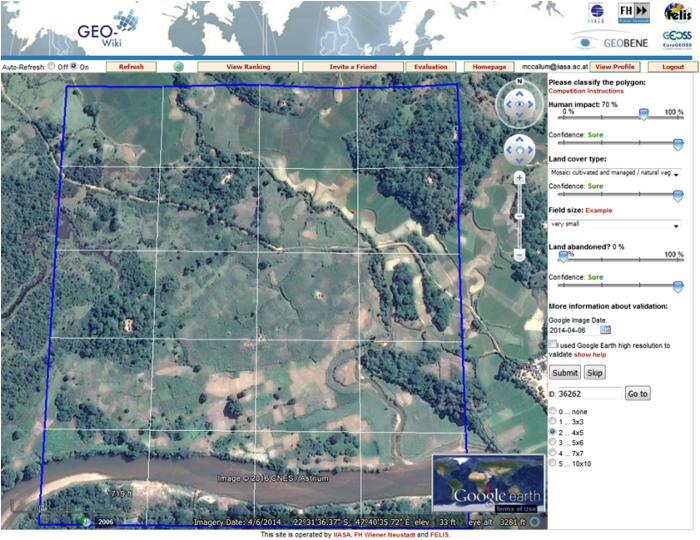
Screenshot of the human impact interface in Geo-Wiki, with the right hand menu filled out according to what is visible in the blue box.

In particular, the datasets described here provide information on human impact, land cover disagreement, wilderness and reference data. The first campaign evaluated a global map of land availability for biofuel production, collecting data on land cover and human impact^14^ (Fig. 1). The second campaign collected reference data in the areas where the Global Land Cover for the year 2000 (GLC-2000)^15^, the Moderate Resolution Imaging Spectroradiometer (MODIS)^16^ and the Global land Cover (GlobCover)^17^ datasets disagree with one another^18^. The third campaign collected land cover and human impact data in order to determine the location and amount of global wilderness^18^. In the fourth campaign, reference data were collected at the same locations as those used to validate the Finer Resolution Observation and Monitoring of Global Land Cover (FROM-GLC) map^19^.

These data are made available for the scientific community interested in reference data for global satellite-derived products, as well as those interested in monitoring global terrestrial ecosystems in general. In particular, they address a large gap in global land cover and land use data and represent the first global crowdsourcing derived land cover and land use reference dataset. Furthermore, they can be used to produce both entirely new datasets^12^ and new hybrid products^20^, and they form the beginning of an open dataset on land cover and land use, which will be expanded in the LandSense citizen observatory for monitoring land cover, land use and change (http://www.landsense.eu).

## Methods

**Figure 2:**
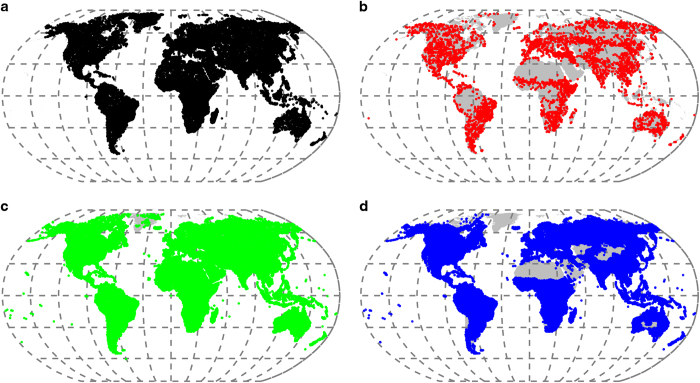
Locations of data collected from the four campaigns. (**a**) Human impact, (**b**) disagreement; (**c**) wilderness; and (**d**) reference.

The global crowdsourcing derived dataset for land cover and land use is comprised of four campaigns: human impact, land cover disagreement, wilderness and reference (Data Citation 1). Although related, the campaigns were underpinned by different research questions so the result has been the acquisition of different geographically distributed data sets that are relevant not only for land cover and land use science but also for the creation of remotely-sensed and hybrid products. An overview of the four campaigns is described in detail below (Table 1). Over 150,000 samples of land cover and land use were acquired globally at more than 100,000 unique locations (Fig. 2).

Unless otherwise noted, the following ten land cover/land use classes were used: [1] tree cover; [2] shrub cover; [3] herbaceous vegetation/grassland; [4] cultivated and managed; [5] mosaic of cultivated and managed/natural vegetation; [6] flooded/wetland; [7] urban; [8] snow and ice; [9] barren; and [10] open water. These form a basic description of global land cover and land use, providing a compromise between retrieving enough information while maintaining simplicity for the contributor. Furthermore, these were chosen to be consistent with generalized land cover classes^21^, which allows for comparison of existing land cover products. In particular, we collapsed all tree classes into a single class, added a mosaic class of cultivated and managed/natural vegetation and added a flooded wetland class^12^. This set of ten classes was deemed optimal for retrieval of information from high to medium resolution satellite imagery.

In most campaigns, the top 10 contributors were awarded co-authorship on the resulting scientific publication, with their final rank based on both quantity and quality of their contributions. Additional prizes were awarded to the top three participants. Quality was tested by comparison with expert-derived control data, cross-comparison among participants and consistency checks^22^. For each campaign, a training manual with an interpretation key was supplied for participants to see examples of different land cover types and different degrees of human impact, where the latter was loosely based on the work of Theobald^23^. Contributors were recruited by emails sent to registered Geo-Wiki volunteers, relevant mailing lists and networks (in particular those with students) and through social media^22^. Hence contributors were generally composed of remote sensing experts and postgraduate students with a background in remote sensing or spatial sciences, along with scientists working in a related field.

### Human impact campaign

A campaign to evaluate a map of land availability for biofuel production was undertaken using the Geo-Wiki crowdsourcing platform^14^. A stratified random sample of 32,946 pixels at a resolution of 1 km was extracted from two sources: the original biofuel land availability map^24^ and cropland locations from the FROM-GLC land cover validation dataset. These were then provided randomly to the participants for assessment. For each pixel, the volunteer was asked to indicate the dominant land cover from a set of 10 basic classes. Additionally, volunteers were asked to provide their level of confidence: unsure; less sure; quite sure; and sure. Furthermore, if the volunteer selected a land cover class containing cultivated vegetation, they were prompted to select a field size: very small; small; medium; and large. The volunteer was then asked to indicate the overall degree of human impact (on a scale of 0 to 100%) that was visible from the satellite images in the same 1 km pixel along with their confidence in their choice as above. Human impact in this context refers to the degree to which the landscape has been modified by humans as visible from satellite imagery. In a final interpretation question, volunteers were asked to determine if the land was abandoned along with an indication of confidence as above. Finally they were asked to record the satellite image date that was visible at the bottom of the screen. A total of 299 control pixels were independently assessed by three experts^22^ for quality assessment purposes (Data Citation 2).

### Disagreement campaign

This campaign focused on global land cover disagreement, sampling from areas where global land cover maps disagree^4^, namely GLC-2000, MODIS and GlobCover. This campaign asked volunteers to identify the dominant land cover class within a 300 m GlobCover pixel, using all classes except the mosaic of cultivated and managed/natural vegetation. In addition, they were asked to indicate the percentage of the pixel covered by the chosen land cover. This could then be repeated for a total of three land cover classes. A check box was provided so that volunteers could indicate if more than three land cover types were present in the pixel. Volunteers were then asked to indicate the overall human impact for the pixel and to further indicate their overall confidence in their choice: unsure; less sure; quite sure; and sure. Finally they were asked to record the satellite image date that was visible at the bottom of the screen, in addition to indicating if the imagery was high-resolution. In contrast to the human impact campaign, the presence of abandoned land and related confidence were not recorded (owing in part to the difficulty that participants had with this attribute). A total of 49 control pixels were independently assessed by three experts for quality assessment purposes (Data Citation 3).

### Wilderness campaign

The aim of the wilderness campaign was to collect human impact and land cover information at the same locations used to validate the global FROM-GLC land cover map^19^, where human impact is used as an inverse proxy for wilderness. Data were collected using a 1 km pixel resolution based on the MODIS grid. Using human impact collected from this and all other campaigns, the data were used to create the first global crowdsourced map of human impact^11^. The campaign employed the same methodology and Geo-Wiki interface for data collection as in the disagreement campaign. However human impact was recorded for each land cover class, up to three land cover types. A total of 175 control pixels were then independently assessed by three experts for quality assessment purposes (Data Citation 4).

### Reference campaign

The reference campaign was conducted on the same locations as those used to validate the global FROM-GLC land cover map^19^, with the aim of creating a robust crowdsourced reference dataset for land cover and land use products generally. These points also coincide with the wilderness campaign, and use the same 1 km MODIS pixels. In contrast to the other campaigns, an additional nine students were recruited by the International Food Policy Research Institute (IFPRI) to collect the data. The campaign employed the same methodology as the wilderness campaign with up to three land cover and human impact values recorded for each pixel. The same control points used in the wilderness campaign were reused for this campaign (Data Citation 4).

## Data Records

The resulting data produced from the four campaigns described above are available in a single dataset in csv format in (Data Citation 1). A total of 151,942 records are available. Three additional datasets are provided for quality control, independently assessed by three experts: a total of 299 control pixels are provided for quality assessment purposes of the human impact campaign in (Data Citation 2); a total of 49 control pixels are provided for quality assessment purposes of the disagreement campaign in (Data Citation 3); and a total of 175 control pixels are provided for quality assessment purposes of the wilderness and reference campaign in (Data Citation 4). All of the above files are georeferenced. The associated metadata for each file is found together with the data files.

## Technical Validation

**Figure 3:**
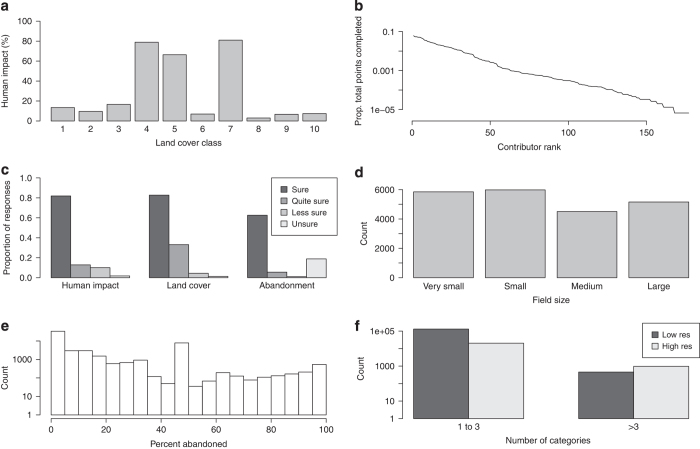
Descriptive plots of key fields found within the campaign dataset comprised of 151,942 records. (**a**) Percent human impact as a function of land cover class. See the metadata records for names of the classes. (**b**) Rank-abundance curve for the work completed by individual contributors to the dataset. (**c**) The relative confidence levels for responses in the three different campaigns where contributors were asked to rate the confidence of their responses. (**d**) Histogram of field sizes from the human impact dataset. (**e**) Histogram of percent abandonment from the human impact dataset. (**f**) The number of categories detected in an image as a function of image resolution.

While the data presented here are raw data, every effort has been made to ensure the data are technically valid. Figure 2 depicts the spatial locations of the four campaigns, displaying both the density of the sampling and the sample design. Figure 3 depicts the key attributes in the dataset and their basic statistics. From Fig. 3a it is clear that land cover classes 4 (cultivated and managed), 5 (mosaic of cultivated and managed/natural vegetation) and 7 (urban) contain values of high human impact as would be expected. In all other land cover classes, lower values of human impact were recorded. Figure 3c shows that confidence is lower for abandoned land, compared with human impact and land cover. Figure 3f provides evidence that if participants recorded more than three land cover categories, they tended to have access to high resolution imagery, while the opposite was shown for less than three land cover categories. Furthermore, technical validation of these data has been reported elsewhere^11,15,25^.

## Usage Notes

These datasets present some of the first global land cover and land use reference datasets obtained via crowdsourcing. In that sense alone they provide a unique dataset from which to study global land cover and land use. Furthermore, they have greatly increased the amount of reference data available to groups developing global biophysical land cover and land use products. It is hoped that by releasing this data, others may find new ways to filter and optimize the usage of crowdsourced land cover and land use data. It is however important to stress that these data are not of high enough quality to be considered as validation data *per se*, and thus we refer to them as reference data.

In particular, these data can be used to understand the quality of crowdsourced data by comparison to external expert measurements, or to built-in measures of quality (redundancy) or to the provided expert control datasets. For example, considering entries with a similar pixelID, it is possible to determine a measure of consistency among the contributors. Furthermore, the userID provides an ability to identify individual users (albeit anonymously) and determine their individual consistency as well as the consistency when compared to others. Additionally, having the transaction time, it is possible to follow the progress of individuals over time. Some of these comparisons have been reported elsewhere^22, 26,27,28^. Clearly, the quality of the data varies among contributors, which is dependent upon several factors including the design of the campaign, e.g. human impact is relatively easier to determine than land cover or land abandonment.

Unlike with human impact, experts were more accurate than non-experts in determining land cover, suggesting that extra training should be provided to those individuals with a non-expert background^22^. A comparison of the control data provided for each campaign (i.e. either of the three expert choices) against the crowdsourced data for the primary land cover class resulted in the following levels of agreement: human impact campaign 66%; disagreement campaign 77%; wilderness campaign 70%; and the reference campaign 72%. In many cases, only Landsat imagery is available which (at 30 m resolution) can make land cover classification challenging. For the disagreement campaign, if only contributions with high resolution imagery (<1 m resolution) are considered, agreement rises to 80%. The lowest accuracies are in shrub cover, grassland/herbaceous and the mosaic cropland class, which indicates the need to provide more examples of how these classes appear on satellite images within the training materials as the volunteers are confusing these classes more often than others^22^.

**Figure 4:**
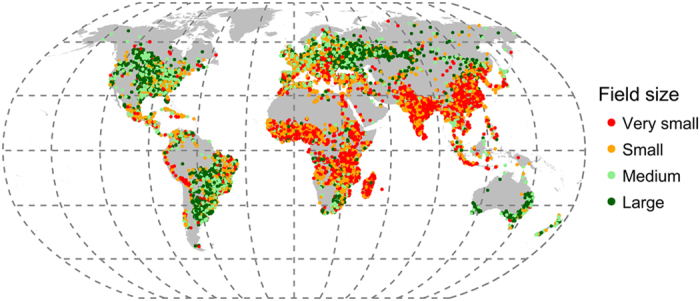
Location and classification of global field size according to contributor response.

Finally, as each data entry contains a geo-coordinate, it is possible to map the data (Fig. 4) and perform various geo-statistical analyses. In particular, these data have been used in the development of new hybrid products^20,29^, and entirely new products derived directly from the crowdsourced datasets themselves^12,18^.

## Additional Information

**How to cite this article:** Fritz, S. *et al.* A global dataset of crowdsourced land cover and land use reference data. *Sci. Data* 4:170075 doi: 10.1038/sdata.2017.75 (2017).

**Publisher’s note:** Springer Nature remains neutral with regard to jurisdictional claims in published maps and institutional affiliations.

## Supplementary Material



## Figures and Tables

**Table 1:** Summary of the main characteristics of the datasets.

**Competition**	**Sample design**	**Resolution**	**Samples**	**Contributors**	**Year**
Human impact	Randomly stratified by land availability for biofuel production and cropland locations via FROM-GLC	1 km MODIS	53,278	65	2011
Disagreement	Stratified by disagreement	300 m GlobCover	30,359	61	2012
Wilderness	Preset, fixed locations based on a globally systematic unaligned sampling strategy	1 km MODIS	32,861	65	2012
Reference	Preset, fixed locations based on a globally systematic unaligned sampling strategy	1 km MODIS	35,444	26	2012
